# Methionine Restriction Partly Recapitulates the Sympathetically Mediated Enhanced Energy Expenditure Induced by Total Amino Acid Restriction in Rats

**DOI:** 10.3390/nu11030707

**Published:** 2019-03-26

**Authors:** Shelby Spring, Arashdeep Singh, Rizaldy C. Zapata, Prasanth K. Chelikani, Adel Pezeshki

**Affiliations:** 1 Department of Animal and Food Sciences, Oklahoma State University, Stillwater, OK 74078, USA; scspring2@wisc.edu; 2 Department of Production Animal Health, Faculty of Veterinary Medicine, University of Calgary, Calgary, AB T2N 4N1, Canada; a.singh@cop.ufl.edu (A.S.); rczapata@ucsd.edu (R.C.Z.); 3 Gastrointestinal Research Group, Snyder Institute for Chronic Diseases, University of Calgary, Calgary, AB T2N 4N1, Canada

**Keywords:** amino acid restriction, methionine restriction, sympathetic system, energy balance, obesity, obesity-prone rats, 6-hydroxydopamine, energy expenditure

## Abstract

Total amino acid (AA) restriction promotes hyperphagia and energy expenditure. We determined whether (i) methionine restriction mimics the effects of total AA restriction, (ii) methionine supplementation attenuates these responses, and iii) sympathetic signaling mediates such effects. Rats were injected with either vehicle (V) or 6-hydroxydopamine (S) to induce chemical sympathectomy, and then randomized to four diets: 16% AA (16AA), 5% AA (5AA), 16% AA-methionine (16AA-Met), and 5% AA+methionine (5AA+Met). Propranolol or ondansetron were injected to examine the role of sympathetic and serotonergic signaling, respectively. 5AA, 5AA+Met, and 16AA-Met increased the food conversion rate for 1–3 weeks in the V and S groups, and increased mean energy expenditure in V group,; the magnitude of these changes was attenuated in the S group. Propranolol decreased the energy expenditure of V16AA, V5AA, and V5AA+Met and of S5AA, S5AA+Met, and S16AA-Met, whereas ondansetron decreased the energy expenditure in only the S groups. Compared to 16AA, the other V groups had reduced body weights from days 7–11 onwards and decreased lean masses throughout the study and the other S groups had decreased body weights and lean masses from day 14 onwards. Total AA restriction enhanced the energy expenditure and reduced the weight and lean mass; these effects were partly recapitulated by methionine restriction and were sympathetically mediated.

## 1. Introduction

In humans, low protein diets have been associated with lower mortality and protection against cancer, diabetes, and chronic kidney disease [1,2]. Similarly, diets that are low in protein and high in carbohydrates have been associated with increased lifespan and improved cardiometabolic health in rodent models [1,3,4,5]. A meta-analysis of 38 experimental human trials found that protein intake was negatively associated with caloric intake, such that the reduction of dietary protein from 20% to 10% markedly increased the energy intake [6]. Consistent with these findings and the protein leverage hypothesis [7], we [8] and others [9,10,11,12,13] have shown that moderate dietary protein restriction (5–10% of total energy) increases energy intake as well as energy expenditure in rodents [8,14,15,16,17,18]. The enhanced energy intake and energy expenditure are often accompanied by a reduction in body weight and body lean mass [6,8,14,18,19] with either an increase [4,6,18,19] or no change in body fat mass [8,14,20,21]. The hyperphagic and thermogenic effects of low protein diets appear to be mediated by multiple mechanisms, which include gut-derived neurohumoral signals and central sensing mechanisms [8,14,15,17,18,22,23,24,25]. Dietary protein restriction reduces the circulating concentrations of most amino acids (AA), and imbalances in these AA activate the general control nonderepressible 2 (GCN2) pathway in the anterior piriform cortex, with projections to the hypothalamus, to induce behavioral responses including early meal termination, foraging, and aversion, which together decrease food intake [14,22,23,24,26]. For example, low protein diets increase circulating histidine and brain histamine concentrations that mediate anorexia [27], and leucine deprivation activates hypothalamic AA sensing and signaling mechanisms to decrease food intake [28]. In contrast, despite a reduction in concentrations of branched-chain amino acids (BCAA: leucine, isoleucine and valine) in circulation [8] and in the brain [22] with protein dilution, these AA were not found to be essential for the hyperphagic effects of low protein diets [29]. Therefore, the underlying mechanisms by which a dietary restriction of protein or AA modulates food intake and energy expenditure to alter body composition are not completely understood.

Among the essential AA, there is substantial evidence that the restriction of methionine plays a role in the metabolic adaptations to low protein diets. Methionine restriction has been shown to increase energy expenditure [13,30,31,32], decrease absolute food intake [30,33,34] or increase absolute food intake and intake per unit of body weight [13,31,35], improve glucose metabolism and cardiac function, decrease oxidative stress, protect against cancer, and promote longevity in rodents [36]. A dietary restriction of methionine and the associated AA imbalance has been reported to influence energy balance, decrease fecundity, and increase longevity in Drosophila [37,38]. Interestingly, methionine supplementation alone to a restricted diet was found to restore fecundity in Drosophila [38]. Similar to the effects of protein restriction on energy expenditure, the increased energy expenditure with a methionine restriction has been associated with enhanced secretion of hepatic fibroblast growth factor-21 [13,39,40], the increased expression of brown fat mitochondrial uncoupling protein-1 [13,31], and the enhanced sympathetic signaling through β-adrenergic receptors (β-AR), particularly in brown fat [32]. The chronic administration of the β1 and β2-AR antagonist, propranolol, to β3 receptor knockout mice has been shown to attenuate the increase in food intake and energy expenditure with methionine-restricted diets [32]. Also, the thermogenic effects of methionine restriction are lost in uncoupling protein-1 knockout mice [13], supportive of a role for β-adrenergic and uncoupling protein-1 signaling mechanisms in the thermogenic effects of dietary methionine restriction. However, it is unknown whether total chemical sympathectomy vitiates the hyperphagic and thermogenic effects of dietary restriction of methionine or other AA. We previously reported that the peripheral 5-hydroxytryptamine (5HT3) receptor signaling, in part, mediates the thermogenic effects of low protein diets [8]. However, it is unknown whether 5HT3 signaling is essential for the hyperphagic and thermogenic effects of methionine-restricted diets.

We previously reported that diets containing 5% protein decreased the plasma concentrations of methionine and BCAA, together with hyperphagia and energy expenditure in rats [8]. There is some evidence that the metabolic responses to dietary protein dilution such as reductions in body weight and fat mass in obese rodent models are mimicked by a restriction of BCAA [41,42,43], yet the replacement of BCAA to protein diluted diets did not reverse the metabolic benefits of protein dilution on glucose metabolism [44]. However, little is known of whether restricting dietary methionine to amounts observed with 5% protein diet mimics the effects of total protein restriction on energy balance. Though methionine supplementation alone to a restricted diet was found to restore fecundity in Drosophila [38], it is unknown whether the supplementation of methionine alone partially annuls the effects of low protein diets on energy balance. The neurotoxin 6-hydroxydopamine hydrobromide (6-OHDA) was often utilized to ablate primarily noradrenergic nerve terminals [45]; however, 6-OHDA-induced chemical sympathectomy has not been applied to investigate the role of sympathetic signaling in the metabolic effects of dietary protein or AA restriction. Therefore, the objectives of the current study were to investigate (1) whether dietary methionine restriction simulates the effects of total AA restriction on energy intake, energy expenditure, and body composition; whether methionine supplementation attenuates the effects of total AA restriction on energy balance; and (2) whether chemical sympathectomy with 6-OHDA, the antagonism of β1 and β2 adrenergic receptors, or the blockade of peripheral 5HT3 signaling negates the effects of total AA restriction or methionine restriction on energy balance in an obesity-prone rat model of the human condition. To assess whether the dietary interventions reduce gains in weight and adipose mass, and given prior evidence that AA restriction reduces adiposity in obese rodent models [41,42,43], rats prone to obesity and fed high-fat obesogenic diets were studied here.

## 2. Materials and Methods 

### 2.1. Animals and Housing 

**Figure 1:**
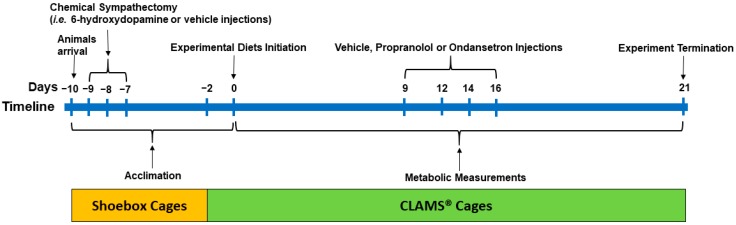
The experimental timeline: Male obesity-prone rats (6 weeks old) were acclimated to the environmental conditions for 8 days in shoebox cages and 2 days in CLAMS^®^ (Comprehensive Lab Animal Monitoring System) upon arrival with continuous access to a high-fat control diet (4.4 kcal/g; 33% fat calories; 16% AA). After arrival (day −10), the animals (*n* = 60) were weight-matched and randomized to two groups to receive injections of either vehicle (V, *n* = 28) or 6-hydroxydopamine to induce sympathectomy (S, *n* = 32) on days −9, −8, and −7 and continued on a high-fat diet for 7 days (i.e., until day 0). At 2 days prior to the initiation of the experimental diet (day −2), the rats were then transferred to CLAMS^®^. The rats within the S and V groups were then randomized to receive four (*n* = 8/diet for S and *n* = 7/diet for V rats) high-fat diets containing 16% AA (control; S16AA and V16AA; 0.46% D,L-methionine w/w as fed), 5% AA (S5AA and V5AA; 0.15% D,L-methionine), 16% AA-methionine (S16AA-Met and V16AA-Met; 0.15% D,L-methionine), or 5% AA+methionine (S5AA+Met and V5AA+Met; 0.46% D,L-methionine) for 21 days. All rats received injections of propranolol, ondansetron, or saline on days 9, 12, 14, and 16 to study the role of sympathetic and serotonergic signaling on energy balance. Metabolic measurements including daily food intake and energy expenditure and weekly body weight and body composition were recorded for 21 days while the rats were individually housed in CLAMS^®^.

The procedures for the experiments were approved by the Animal Care Committee of the University of Calgary (AC12-0033). Sixty male obesity-prone Sprague Dawley rats (approx. 210 g, 6 weeks old; Crl: OP-CD, Strain 463; Charles River, Montreal, QC, Canada) were used in this study. Upon arrival in our facility, the animals were acclimated to the environmental conditions for 8 days (10 hours light-dark cycle; 23–24 °C; humidity of 21–22%) in shoebox cages and 2 days in CLAMS^®^ (Comprehensive Lab Animal Monitoring System, Columbus Instruments; Columbus, OH, USA) cages and remained in the CLAMS^®^ till termination on day 21. The animals had ad libitum access to fresh food and water throughout the study period. The experimental timeline is outlined in Figure 1.

### 2.2. Chemical Sympathectomy 

After arrival (day −10), the rats (*n* = 60) were provided with a high-fat control diet (4.4 kcal/g; 33% fat calories and 16% AA; Table 1). In our previous studies, a 33% fat diet was used to induce obesity in obesity prone rats [8,46], and hence, a similar fat content was included in the present study. The rats were weight-matched and randomized to two groups to receive intraperitoneal injections of either vehicle (0.5 mL; 0.1% ascorbic acid in sterile saline; *n* = 28, 201 g ± 2 body weight) or 6-OHDA (Sigma-Aldrich, Oakville, ON, Canada, #H4381; *n* =32, 214 g ± 2 body weight) at 40 mg/kg body weight on day −9 and 80 mg/kg body weight on days −8 and −7 (0.5 mL; 0.1% ascorbic acid in sterile saline), respectively (Figure 1). The dosage of 6-OHDA was based on previous literature [47]. To minimize confounds of drug-induced anorexia and weight loss with our dietary interventions, the rats were adapted to the experimental conditions for 7days (5 days in shoebox cages and 2 days in CLAMS^®^) following the last day (day −7) of vehicle or 6-OHDA injections, while continuing to receive a high-fat control diet until start of the experimental diets (day 0).

### 2.3. Experimental Diets

At 2 days prior to the initiation of the experimental diet (day −2), the sympathectomized (S) and vehicle treated (V) rats were then individually housed in CLAMS^®^ (Figure 1). The rats within the S and V groups were weight-matched and randomized to four isocaloric high-fat diets (*n* = 8/diet for S and *n* = 7/diet for V rats; 4.4 kcal/g; 33% fat calories) containing either 16% AA (control; S16AA and V16AA; 0.46% D,L-methionine w/w as fed), 5% AA (total AA restriction; S5AA and V5AA; 0.15% D,L-methionine), 16% AA-methionine (methionine restriction; S16AA-Met and V16AA-Met; 0.15% D,L-methionine), or 5% AA+methionine (methionine supplementation; S5AA+Met and V5AA+Met; 0.46% D,L-methionine). The S and V rats were fed the respective treatment diets from day 0 to day 21 in CLAMS^®^. We previously reported that diets containing 5% protein consistently increased energy intake and energy expenditure [8]; hence, 5AA was selected for the current study. The 16AA-Met diet was formulated to contain the same amount of methionine as 5AA, while 5AA+Met was formulated to contain the same concentration of methionine as 16AA. Dietary protein was provided in the form of AA mixtures formulated following AIN-93 recommendations [48]. The diets were prepared in-house (Table 1) with ingredients obtained from Dyets Inc. (Bethlehem, PA, USA).

### 2.4. Metabolic Measurements

Food intake and energy expenditure were measured daily (20 h per day) using the CLAMS^®^ as we previously reported [8,46]. The body composition was measured weekly (Minispec LF110^®^ NMR Analyzer; Bruker Corporation, Milton, ON, Canada), and the body weight was recorded twice a week. The general husbandry and maintenance were carried out as we described earlier [8,46]. 

### 2.5. Blockade of β-AR and 5HT3 Receptors 

To assess the role of β-AR and 5HT3 receptors in mediating the effects of the test diets on energy balance, propranolol hydrochloride (Sigma-Aldrich, Oakville, ON, Canada, #P8688), a β1 and β2-AR blocker, and ondansetron hydrochloride (Tocris, Burlington, ON, Canada, #2891), a selective 5HT3 receptor antagonist, were used, respectively (Figure 1). The drugs were administered on days 9, 12, 14, and 16 to both vehicle- and 6-OHDA-treated rats. On any given day, following an overnight fast, the rats were randomized to receive: 1) a subcutaneous injection of saline (0.5 mL), 2) a subcutaneous injection of propranolol (0.5 mL; 10 mg/kg body weight in sterile 0.9% saline), 3) an intraperitoneal injection of saline (0.5 mL), or 4) an intraperitoneal injection of ondansetron (0.5 mL; 1 mg/kg body weight in sterile 0.9% saline) 30 min before the onset of the dark period. The rats were fed with their respective experimental diets (i.e., 16AA, 5AA, 5AA+Met, and 16AA-Met) following the drug injections. 

### 2.6. Statistical Analysis

Repeated measures on energy intake, energy expenditure, body weight, food conversion rate (i.e., energy intake to energy deposited ratio), relative energy intake (i.e., energy intake per 100 g body weight), energy efficiency (i.e., energy deposited to energy intake ratio), mean weekly energy intake and energy expenditure, and body composition data were analyzed using a linear mixed model (IBM SPSS Statistics Version 23, Armonk, NY, USA). The overall model included fixed effects of treatment (i.e., 6-OHDA vs. vehicle); diet (16AA, 5AA, 5AA+Met, and 16AA-Met); time; and the interactions of treatment × diet, treatment × time, diet × time, and treatment × diet × time. Within each treatment, the effects of diet, time, and diet × time on metabolic measurements were then analyzed independently. For the energy expenditure analysis, the sum of the lean mass and 0.2 × fat mass was considered as a covariate following a previous report [49]. For the area under the curve (AUC) data on blocker effects (i.e., propranolol and ondansetron), the model for each blocker included the fixed effects of treatment, drug (blocker vs. saline), diet, and the interactions of treatment, drug, and diet. Within each treatment, the effects of diet, drug, and the interactions of diet and drug on energy intake and energy expenditure were then analyzed independently. The animal was considered as a random variable for all analyses. Based on the smallest values of fit statistics for corrected Akaike Information Criterion and Bayesian Information Criterion, the covariance structure of the repeated measurements for each variable was modeled. Means of the diets within vehicle or 6-OHDA at each time point were separated by paired Student’s t-test followed by a Benjamini–Hochberg correction [50] with a false discovery rate of 0.10 for 5 preplanned comparisons, which include 16AA vs. 5AA, 5AA+Met or 16AA-Met, and 5AA vs. 5AA+Met or 16AA-Met. For the AUC data on blocker effects within each treatment, dietary group, and time (dark vs. light), a paired t test was used to separate the means of blocker vs. saline. The data were presented as the mean ± standard error of the mean (SEM). *p* < 0.05 was considered a declaration of a significant difference, and trends were indicated at *p* < 0.10.

## 3. Results

### 3.1. Energy Intake and Body Composition

**Figure 2:**
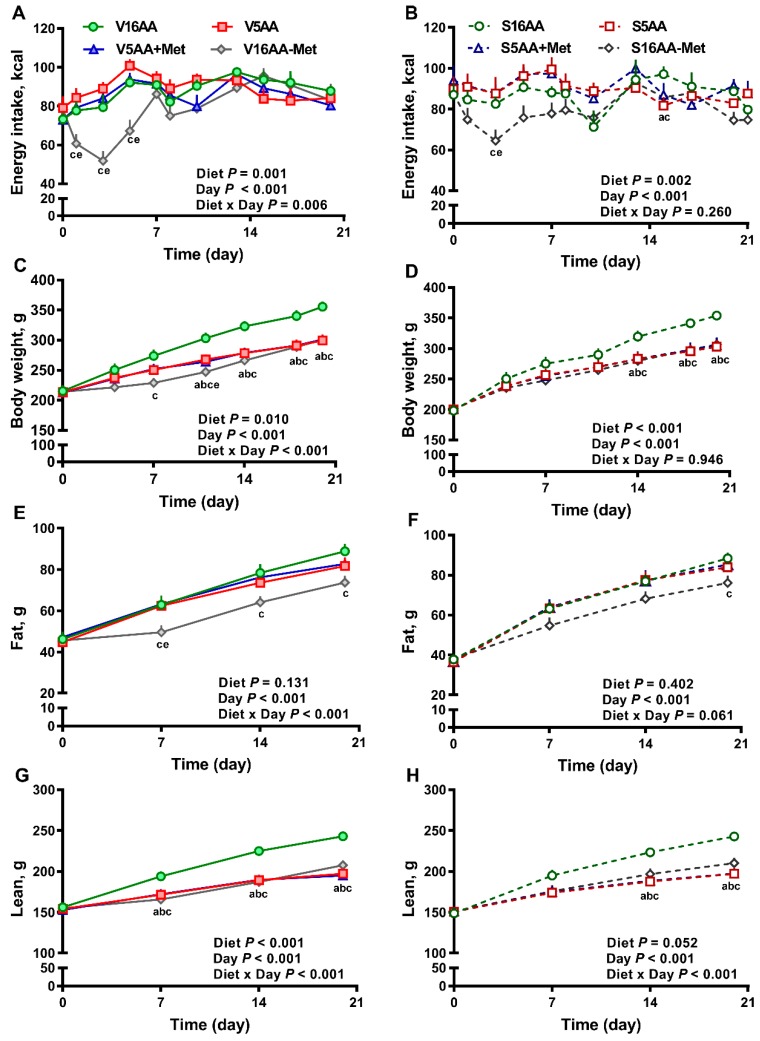
The effect of dietary methionine restriction or supplementation on energy intake (**A**,**B**), body weight (**C**,**D**), body fat mass (**E**,**F**), and body lean mass (**G**,**H**) of vehicle- (**A**,**C**,**E**,**G**) and 6-hydroxydopamine- (6-OHDA) (**B**,**D**,**F**,**H**) treated obesity-prone rats during the 3-week study: The data for the antagonists’ administration days (i.e., days 9, 12, 14, and 16) were not included in the graphs or data analysis. The *p*-values for the overall model effects of treatment, diet, day, diet × day, and treatment × day for energy intake were 0.35, <0.01, <0.01, <0.01, and 0.01, respectively; for body weight were 0.43, <0.01, <0.01, and 0.04, respectively; for body fat were 0.78, 0.04, <0.01, <0.01, and <0.01; and for body lean were 0.77, <0.01, <0.01, <0.01, and <0.01, respectively. The nonsignificant (*p* > 0.05) interactions are not shown. V16AA, vehicle-treated 16% amino acid diet; V5AA, vehicle-treated 5% amino acid diet; V5AA+Met, vehicle-treated 5% amino acid and methionine-supplemented diet; V16AA-Met, vehicle-treated 16% amino acids and methionine-restricted diet; S16AA, sympathectomized 16% amino acid diet; S5AA, sympathectomized 5% amino acid diet; S5AA+Met, sympathectomized 5% amino acids and methionine-supplemented diet; S16AA-Met, sympathectomized 16% amino acids and methionine-restricted diet. For each variable within each treatment and day, ^a^ *p* ≤ 0.05 16AA vs. 5AA, ^b^ *p* ≤ 0.05 16AA vs. 5AA+Met, ^c^ *p* ≤ 0.05 16AA vs. 16AA-Met, and ^e^ *p* ≤ 0.05 5AA vs. 16AA-Met. The values are the mean ± SEM, *n* = 7–8.

**Figure 3:**
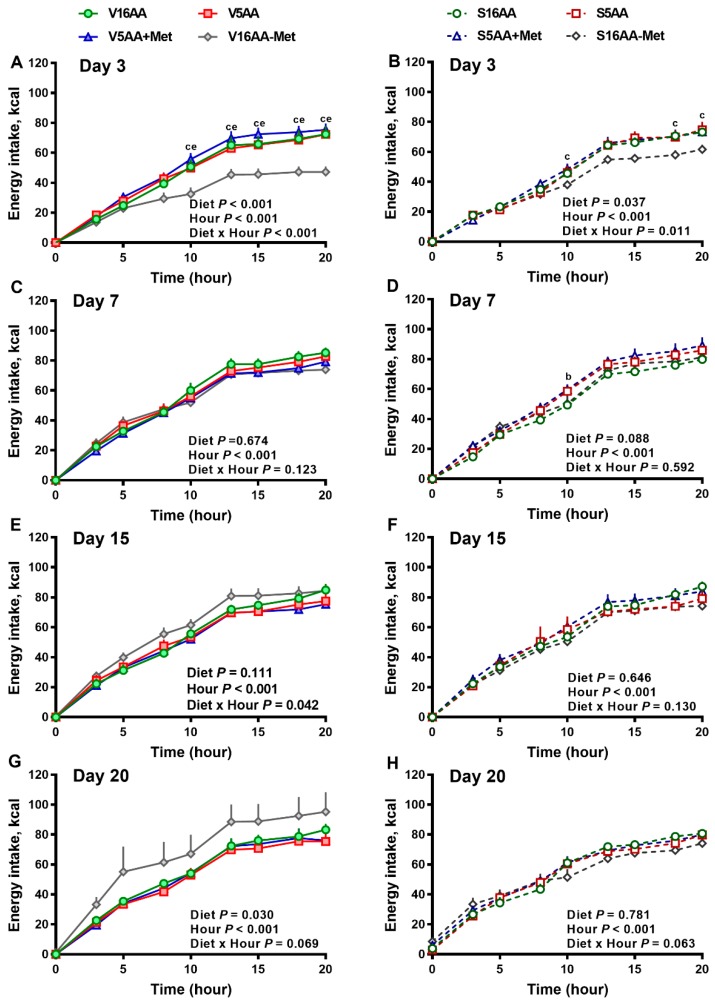
The effect of dietary methionine restriction or supplementation on the energy intake of vehicle- (**A**,**C**,**E**,**G**) and 6-hydroxydopamine- (6-OHDA) (**B**,**D**,**F**,**H**) treated obesity-prone rats on day 3 (**A**,**B**), 7 (**C**,**D**), 15 (**E**,**F**), and 20 (**G**,**H**): The data for the antagonists’ administration days (i.e., days 9, 12, 14, and 16) were not included in the graphs or data analysis. The first 10 h are the dark period. The *p*-values for the overall model effects for treatment, diet, hour, and treatment × diet for energy intake on day 3 were <0.01, <0.01, <0.01, and 0.06, respectively; on day 7 were 0.89, 0.26, <0.01, and <0.01, respectively; on day 15 were 0.81, 0.11, <0.01, and <0.01, respectively; and on day 20 were 0.19, <0.01, <0.01, and <0.01, respectively. The nonsignificant (*p* > 0.05) interactions (i.e., diet × hour, treatment × hour, and treatment × diet × hour) for some days are not shown. V16AA, vehicle-treated 16% amino acid diet; V5AA, vehicle-treated 5% amino acid diet; V5AA+Met, vehicle-treated 5% amino acid and methionine-supplemented diet; V16AA-Met, vehicle-treated 16% amino acids and methionine-restricted diet; S16AA, sympathectomized 16% amino acid diet; S5AA, sympathectomized 5% amino acid diet; S5AA+Met, sympathectomized 5% amino acids and methionine-supplemented diet; and S16AA-Met, sympathectomized 16% amino acids and methionine-restricted diet. For each treatment and day at each hour, ^b^ *p* ≤ 0.05 16AA vs. 5AA+Met, ^c^ *p* ≤ 0.05 16AA vs. 16AA-Met, and ^e^ *p* ≤ 0.05 5AA vs. 16AA-Met. Values are mean ± SEM, *n* = 7–8.

The overall model effects for diet (*p* < 0.01) and diet × day (*p* < 0.05) were significant for energy intake (Figure 2 and Figure 5). In the vehicle group, during the first week, the daily energy intake of V16AA-Met rats was 15% and 23% lower (*p* < 0.05) compared with the intake of V16AA or V5AA rats, respectively (Figure 2A, Figure 3A). Although the daily energy intake did not differ between V16AA and V5AA, the mean weekly energy intake of V5AA was increased compared to V16AA by 11% (*p* < 0.05) in the first week (Figure 5A). In the 6-OHDA group, the daily energy intake of S16AA-Met was lower than S16AA and S5AA on day 3 (*p* < 0.05); the other groups did not differ (Figure 2B, Figure 3B). The mean weekly energy intake of S5AA was 14% higher than S16AA in the first week, and the energy intake of S16AA-Met was lower than S16AA and S5AA by 16% and 26% (*p* < 0.05), respectively (Figure 5B). Thus, although the overall energy intake of the 6-OHDA and vehicle groups did not differ (*p* = 0.35), the transient hyperphagic responses to 5AA and the hypophagic effects of 16AA-Met were preserved in both the vehicle- or 6-OHDA-treated groups. 

Though the body weights of the vehicle- and 6-OHDA-treated groups did not differ (*p* = 0.43), in the vehicle group, compared to V16AA, the body weight of the V16AA-Met rats was decreased from day 7 onwards resulting in an approx. 16% decrease by day 21, and the body weight of the V5AA and V5AA+Met rats were similarly decreased from day 11 onwards leading to an 11% decrease (*p* < 0.05; Figure 2C). In the 6-OHDA group, relative to S16AA, the body weights of S16AA-Met, S5AA, and S5AA+Met were decreased after day 14, resulting in a 13% reduction by day 21 (*p* < 0.05; Figure 2D). Thus, a restriction of the total AA or methionine alone decreased the body weight in both the vehicle- and 6-OHDA-treated groups. 

In the vehicle group, compared to V16AA, food conversion rate (i.e., the ratio of energy intake to energy deposited) was increased for V5AA and V5AA+Met by 53% and 44%, respectively, during the first two weeks and for V16AA-Met by 204% during the first week of the study (*p* < 0.05; Table 2). As expected, the energy efficiency (i.e., ratio of energy deposited to energy intake) was decreased for V5AA and V5AA+Met during the first two weeks and for V16AA-Met during the first week of the study when compared to V16AA (Table 2). When energy intake was normalized to 100 g body weight, compared to V16AA, the relative energy intake was increased in V5AA and V5AA+Met by 13% and 9%, respectively, in the first week of study, and for V16AA-Met, it was increased by approx. 24% during the second week of the study (*p* < 0.05; Table 2). Within the 6-OHDA group, relative to S16AA, food conversion rate was increased for S5AA, S5AA+Met, and S16AA-Met by 47%, 52%, and 33% respectively, during the entire study (*p* < 0.05; Table 2). The energy efficiency of S5AA, S5AA+Met, and S16AA-Met was decreased during the entire study when compared to S16AA (Table 2). Compared to S16AA, the relative energy intake (i.e., energy intake/100 g body weight) was increased in S5AA and S5AA+Met by 20% and 18%, respectively, during the first week of the study, and for S5AA+Met by approx. 17% in the third week of the study (*p* < 0.05; Table 2). Thus, the restriction of the total AA or methionine alone comparably decreased the efficiency of energy deposition in both the vehicle- and 6-OHDA-treated groups. 

The body fat mass (*p* = 0.78) and body lean mass (*p* = 0.77) of the 6-OHDA and vehicle groups did not differ. However, in the vehicle group, compared to 16AA, the body fat mass of V16AA-Met was lower by 16% throughout the study (*p* < 0.05); the other diets did not differ (Figure 2E). In the 6-OHDA group, compared to S16AA, the body fat mass was decreased by 14% in S16AA-Met only at the end of study (*p* < 0.05); the other groups did not differ (Figure 2F). Relative to V16AA, the body lean mass was decreased by 13% in V16AA-Met, V5AA, and V5AA+Met throughout the study (*p* < 0.05; Figure 2G). Compared to S16AA, the body lean mass was significantly decreased by 13%, 18%, and 17%, respectively, in S16AA-Met, S5AA, and S5AA+Met from day 14 onwards (*p* < 0.05; Figure 2H). Thus, overall, the reductions in body fat and lean mass with a total AA restriction were comparable to those of methionine restriction alone, with these effects being delayed and greatly attenuated with 6-OHDA treatment.

### 3.2. Energy Expenditure

**Figure 4:**
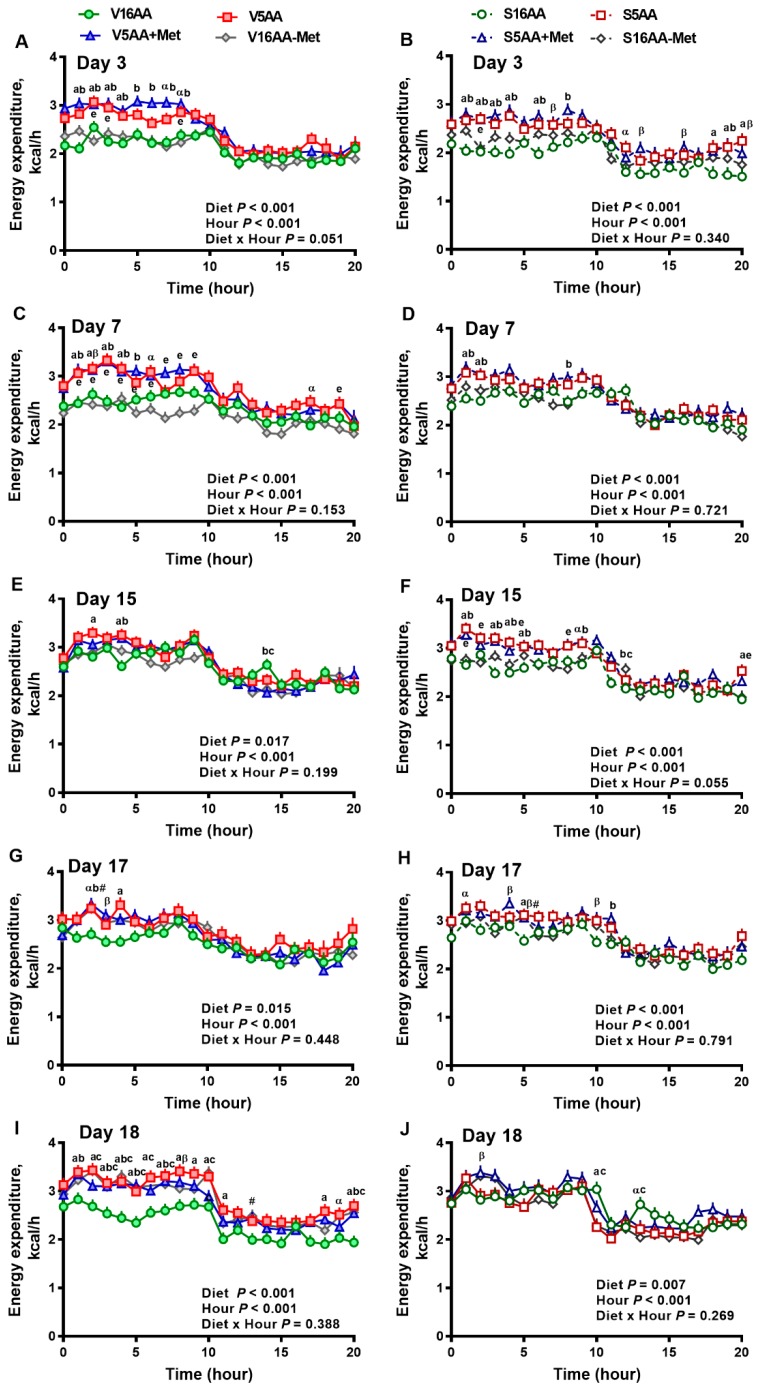
The effect of dietary methionine restriction or supplementation on the energy expenditure of vehicle- (**A**,**C**,**E**,**G**,**I**) and 6-hydroxydopamine- (6-OHDA) (**B**,**D**,**F**,**H**,**J**) treated obesity-prone rats on days 3 (**A**,**B**), 7 (**C**,**D**), 15 (**E**,**F**), 17 (**G**,**H**), and 18 (**I**,**J**): The data for the antagonists’ administration days (i.e., days 9, 12, 14, and 16) were not included in the graphs or data analysis. The first 10 hours are the dark period. The *p*-values for the overall model effects for treatment, diet, hour, diet × hour, diet × treatment, and treatment × hour for energy expenditure on day 3 were <0.01, <0.01, <0.01, 0.01, 0.47, and 0.55, respectively; for day 7 were 0.92, <0.01, <0.01, 0.05, <0.01, and 0.81, respectively; for day 15 were 0.22, <0.01, <0.01, 0.02, <0.01, and <0.01, respectively; for day 17 were 0.55, <0.01, <0.01, 0.61, 0.04, and 0.46, respectively; and for day 18 were 0.72, <0.01, <0.01, 0.05, <0.01, and <0.01, respectively. The nonsignificant (*p* > 0.05) interactions are not shown. V16AA, vehicle-treated 16% amino acid diet; V5AA, vehicle-treated 5% amino acid diet; V5AA+Met, vehicle-treated 5% amino acid and methionine-supplemented diet; V16AA-Met, vehicle-treated 16% amino acid and methionine-restricted diet; S16AA, sympathectomized 16% amino acid diet; S-5AA, sympathectomized 5% amino acid diet; S5AA+Met, sympathectomized 5% amino acids and methionine-supplemented diet; S16AA-Met, sympathectomized 16% amino acid and methionine-restricted diet. For each treatment and day at each hour, ^a^ *p* ≤ 0.05 16AA vs. 5AA, ^b^ *p* ≤ 0.05 16AA vs. 5AA+Met, ^c^ *p* ≤ 0.05 16AA vs. 16AA-Met, ^e^ *p* ≤ 0.05 5AA vs. 16AA-Met, ^α^ *p* ≤ 0.10 16AA vs. 5AA, ^β^ *p* ≤ 0.10 16AA vs. 5AA+Met, and ^#^ *p* ≤ 0.10 16AA-Met vs. 16AA. The values are mean ± SEM, *n* = 7–8.

**Figure 5:**
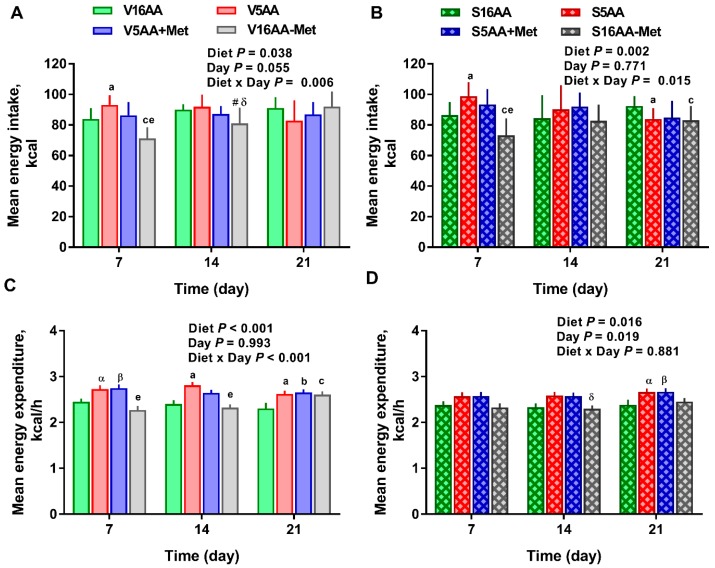
The effect of dietary methionine restriction or supplementation on mean weekly energy intake (**A**,**B**) and mean weekly energy expenditure (**C**,**D**) of the vehicle- (**A**,**C**) and 6-hydroxydopamine- (**B**,**D**) treated obesity-prone rats: The data for the antagonists’ administration days (i.e., days 9, 12, 14, and 16) were not included in the graphs or data analysis. The *p*-values for the overall model effects for treatment, diet, day, and diet × day for energy intake were 0.49, <0.01, 0.44, and 0.01, respectively, and for energy expenditure were 0.10, <0.01, 0.35, and 0.04, respectively. The nonsignificant (*p* > 0.05) interactions are not shown. V16AA, vehicle-treated 16% amino acid diet; V5AA, vehicle-treated 5% amino acid diet; V5AA+Met, vehicle-treated 5% amino acid and methionine-supplemented diet; V16AA-Met, vehicle-treated 16% amino acid and methionine-restricted diet; S16AA, sympathectomized 16% amino acid diet; S-5AA, sympathectomized 5% amino acid diet; S5AA+Met, sympathectomized 5% amino acids and methionine-supplemented diet; S16AA-Met, sympathectomized 16% amino acid and methionine-restricted diet. For each variable within each treatment and day, ^a^ *p* ≤ 0.05 16AA vs. 5AA, ^b^ *p* ≤ 0.05 16AA vs. 5AA+Met, ^c^ *p* ≤ 0.05 16AA vs. 16AA-Met, ^e^ *p* ≤ 0.05 5AA vs. 16AA-Met, ^α^ *p* ≤ 0.10 16AA vs. 5AA, ^β^ *p* ≤ 0.10 5AA+Met vs. 16AA, ^#^ *p* ≤ 0.10 16AA-Met vs. 16AA, and ^δ^ *p* ≤ 0.10 5AA vs. 16AA-Met. The values are the mean ± SEM, *n* = 7–8.

The overall model effects for diet (*p* < 0.01) and diet × day (*p* < 0.05) were significant, and treatment tended (*p* < 0.10) to be significant with the mean energy expenditure being greater in the vehicle- (2.55 ± 0.03) vs. 6-OHDA- (2.48 ± 0.02) treated rats (Figure 4 and Figure 5). In the vehicle group, relative to V16AA, the energy expenditure of V5AA and V5AA+Met was increased by 20% and 24%, respectively, during the dark period of the first week (*p* < 0.05; Figure 4A,C). Compared to the V16AA, the energy expenditure of rats consuming the V5AA, V5AA+Met, and V16AA-Met diets increased by 44%, 39%, and 40%, respectively, during both the dark and light periods from second week onwards (*p* < 0.05; Figure 4G,I). The energy expenditure of V16AA-Met was higher than V16AA on day 17 and 18 during the dark period (*p* < 0.05; Figure 4 G,I). When the data were analyzed on a weekly basis, relative to V16AA, V5AA increased energy expenditure during weeks 1 to 3 but V5AA+Met increased the mean energy expenditure during week 1 and 3 and V16AA-Met only increased energy expenditure in week 3 (*p* < 0.05; Figure 5C). In the 6-OHDA group, relative to V16AA, the energy expenditure of V5AA and V5AA+Met was increased by 17% and 21%, respectively, during the dark period of the first week (*p* < 0.05; Figure 4B,D). In the 6-OHDA treated animals, relative to S16AA, the energy expenditure of S5AA, S5AA+Met, and S16AA-Met was increased by 12%, 14%, and 5%, respectively, from the second week onwards (Figure 4H,J). The energy expenditure of S16AA-Met only showed transient increases compared to S16AA on day 15 and 17 (Figure 4F,H) but did not differ on day 18 (Figure 4J). The weekly energy expenditure analysis revealed that compared to S16AA, S5AA and S5AA+Met tended to increase energy expenditure but S16AA-Met did not differ during week 3 (*p* < 0.05; Figure 5D). Within the vehicle- or 6-OHDA-treated groups, energy expenditure did not differ between 5AA and 5AA+Met. Thus, overall, the restriction of total AA or methionine alone enhanced energy expenditure in vehicle-treated rats, but the mean energy expenditure was lower and the magnitude of increases by the AA-restricted diets were attenuated and delayed by 6-OHDA treatment. 

### 3.3. β-AR Blockade with Propranolol

Within the vehicle group, relative to saline, the AUC for energy intake in V5AA+Met decreased during the dark period by about 24% following the administration of propranolol (*p* < 0.05), whereas V16AA, V5AA, and V16AA-Met did not differ (Table 3). Relative to saline, propranolol decreased the energy expenditure of V16AA, V5AA, and V5AA+Met by approx. 12%, 18%, and 12%, respectively, during the dark period (*p* < 0.05; Table 3). In the 6-OHDA group, compared to saline, propranolol decreased the energy intake of S5AA, S5AA+Met, and S16AA-Met during the dark cycle by about 18%, 28%, and 30%, respectively (*p* < 0.05; Table 3). Relative to saline, propranolol did not affect the energy expenditure of S16AA but decreased the energy expenditure of the S5AA, S5AA+Met, and S16AA-Met groups by 32%, 23%, and 21%, respectively, during the dark period (*p* < 0.05 for S5AA and S5AA+Met and *p* = 0.09 for S16AA-Met; Table 3). Thus, β–AR blockade with propranolol decreased energy expenditure comparably in the total AA and methionine-restricted rats regardless of 6-OHDA treatment. 

### 3.4. HT3 Receptor Blockade with Ondansetron

In the vehicle and 6-OHDA groups, within the various diets, no differences in the energy intake of animals administered with ondansetron vs. saline were detected during the dark period (Table 4). In the vehicle group, no differences in the energy expenditure was detected between rats administered with ondansetron vs. saline, but within the 6-OHDA group, ondansetron decreased the energy expenditure of the S16AA, S5AA, S5AA+Met, and S16AA-Met groups by 23%, 21%, 18%, and 19%, respectively, during the dark period (*p* < 0.05; Table 4). Overall, the energy intake and energy expenditure did not differ between the 6-OHDA and vehicle groups.

## 4. Discussion

We previously reported that diets containing 5% protein decreased circulating methionine concentrations, promoted hyperphagia and energy expenditure, and decreased weight gain in rats [8]. Here, we addressed whether such effects could be reproduced by restricting methionine alone, whether they could be reversed by methionine supplementation, and whether sympathetic signaling plays a role in mediating such responses. Our data revealed several important findings. First, total AA restriction transiently increased energy intake during the first week in both vehicle and 6-OHDA rats, and further, total AA and methionine restrictions induced an increase in the food conversion rate and decreased the efficiency of energy deposition during the first two weeks in vehicle and all three weeks in 6-OHDA treated rats. This suggests that sympathetic innervation is not essential for the hyperphagic responses to total AA restriction and for the reduction in energy efficiency with total AA or methionine restriction. Second, sympathetic denervation with 6-OHDA decreased the overall mean energy expenditure. Notably, the enhanced energy expenditure induced by total AA and methionine restrictions were attenuated in 6-OHDA-treated rats, indicating that the energy expended with these diets were in part sympathetically mediated. Third, propranolol decreased energy expenditure in total AA-restricted and in methionine-restricted and supplemented diets in 6-OHDA-treated rats, indicating that β1 and β2 adrenergic signaling was important for the enhanced energy expenditure with these diets. Though 5HT3 receptor signaling was not essential for the effects of the diets on energy intake, a higher serotonergic tone via 5HT3 signaling appears to be important for mediating the effects of the diets on energy expenditure in 6-OHDA-treated rats. Fourth, total AA and methionine restrictions decreased body weight and lean mass, with methionine restriction also decreasing adipose mass. These changes in body composition were delayed in 6-OHDA-treated rats, suggesting that sympathectomy protected against the diet-induced changes in the tissue compartments. Thus, dietary methionine restriction partly recapitulated the enhanced energy expenditure, weight, and lean loss resulting from total AA restriction, which was likely through enhanced sympathetic signaling, whereas the methionine restriction-induced increase in transient hyperphagia and reduction in energy efficiency were independent of sympathetic signaling.

Here, we show that the 5AA diets induced hyperphagia, which was generally similar to previous reports showing that protein-diluted diets promote hyperphagia in rats, mice, and humans [8,9,10,11,12,52]. Since the initial increase in the food intake of 5AA+Met was comparable to 5AA, it suggests that methionine supplementation alone was insufficient to partially reverse the hyperphagic response to protein dilution. Further, V-16AA-Met significantly decreased the absolute amount of food consumed during the first week, consistent with other reports [30,33,34]. Though we did not use taste aversion tests, it is likely that the initial hypophagic effects of methionine-restricted diets may also be due to a mild aversion or novelty from the amino acid imbalance in the diet. However, we found that rats fed with 5AA and 16AA-Met diets consumed more calories per energy deposited or per 100 g body weight, indicative of decreased feed efficiency, and is comparable to the outcomes of other studies with similar degree (0.12%–0.25% methionine) of methionine restriction [13,31,35]. Less is known of the underlying mechanisms by which dietary protein or amino acid restriction promote hyperphagia. Though we [8] and others [29] have shown that circulating BCAA are decreased with dietary protein restriction, the decreased concentrations of these amino acids in the circulation or brain appear to be insufficient to illicit hyperphagia in rats [29]. Nonetheless, there is substantial evidence indicating that dietary AA insufficiency is sensed by cellular signal transduction mechanisms, which include the activation of the GCN2–eukaryotic translation initiation factor 2 subunit 1 (eIF2α) pathway with a coordinate repression of the mammalian/mechanistic target of rapamycin complex 1 (mTORC1) and the integrated stress response (ISR) pathways to block translation in peripheral tissues [53,54]. Though a similar GCN2–eIF2α signaling in the anterior piriform cortex has been implicated in the feeding behavioral responses to dietary AA imbalance [26], dietary AA reduces the ISR and mTORC1 signaling, and associated protein synthesis in the periphery [55,56,57], the relative importance of these peripheral and central mechanisms that sense methionine deficiency remains largely unknown.

The enhanced energy expenditure with the 5AA, 5AA+Met, and V-16AA-Met diets was consistent with the increased energy expenditure resulting from dietary protein dilution [8,14,15,16,17,18,52] or with methionine restriction [13,30,31,32,58] in rodents. The underlying mechanisms by which total AA restriction or methionine restriction increase energy expenditure are not completely understood. We previously found that diets containing 5% protein decreased plasma methionine concentrations [8], and others reported that a 2% protein diet rapidly decreased the concentrations of methionine in both the plasma and brain [22]; it is unknown whether the depletion of central methionine mediates the metabolic responses to protein dilution. However, in the periphery, protein-restricted diets have been proposed to increase energy expenditure in part via stimulating hepatic fibroblast growth factor 21 secretion and enhancing sympathetic signaling through β-AR and uncoupling protein-1 in brown fat [13,14,15,17,18,25,31,32,35,39,40]. Although the administration of propranolol (β1 and β-2-blocker) to β3-knockout mice attenuated the hyperphagic and thermogenic effects of methionine restriction [32], the effect of total chemical sympathectomy on these metabolic responses has not been investigated to date. We now show that the increased energy expenditure with V-5AA and V-16AA-Met diets were attenuated or lost in the 6-OHDA-treated rats, indicating that chemical sympathectomy partially abrogates the increased energy expenditure of 5AA and 16AA-Met. We also found that the β1 and β2-AR antagonist propranolol decreased the energy expenditure of V-16AA, V-5AA, and V-5AA+Met rats, primarily during the dark period when the activity and intake of rats were at a maximum. Of note, propranolol also produced a significant reduction in energy expenditure in the S-5AA, S-5AA+Met, and S-16AA-Met. Because 6-OHDA treatment increases the protein abundance of β1 and β2-AR in the colon with enhanced sensitivity to dopamine and norepinephrine to colonic ion transport [59], it is likely that the upregulation of peripheral β1 and β2-AR contributed to the robust effects of propranolol on energy expenditure in 6-OHDA-treated rats. Alternatively, it is also likely that 6-OHDA was ineffective in producing a total ablation of noradrenergic nerve terminals with the subsequent recovery of β-adrenergic signaling over the 3-week course of the study. However, 6-OHDA was reported to cause over a 72% reduction in splenic norepinephrine content for a minimum 15 d post-injection in rats [47], a 70% reduction in norepinephrine content in heart and salivary glands as well as nonvascular smooth muscle contraction for a minimum 21 day post-injection in rats [45], and a decrease in tyrosine hydroxylase immunopositive cells in pancreas for up to 30 days post-administration in mice [60]. A potential caveat is that, due to an accidental −80°C freezer malfunction, the tissue norepinephrine content could not be confirmed in the current study. Nonetheless, others [45,47] have clearly established that the 6-OHDA regimen, as used in the current study, decreases tissue norepinephrine for at least 15 days post-injection in rats. Given that the energy intake and energy expenditure responses are, for the most part, indistinguishable between the 5AA and 5AA+Met diets, this indicates that the replenishment of methionine alone is insufficient to completely or partially reverse the metabolic responses to 5AA and is likely due to a deficiency of other limiting amino acids than methionine in the 5AA+Met group.

We previously reported that dietary protein restriction dose-dependently decreased circulating concentrations of tryptophan, a serotonin precursor, and that serotonergic 5HT3 receptor signaling was important for the hyperphagic and thermogenic effects of a 10% protein diet [8]. Prior evidence indicates that dietary tryptophan restriction decreases central and plasma serotonin [61], inhibits growth [62], and modulates energy balance in rodents [63]. Further, dietary restriction of all amino acids and, in particular, methionine alone has been shown to decrease serotonin concentrations in rodent brain [64]. In the present study, ondansetron, a selective 5HT3 receptor blocker, did not change the energy intake, suggesting that serotonergic-5HT3 signaling is unlikely to mediate the effects of 5AA or 16AA-Met on energy intake. However, the ondansetron-induced reduction in energy expenditure in the 6-OHDA groups indicates that peripheral 5HT3 signaling is important for sustaining energy expenditure in sympathectomized rats; whether sympathectomy upregulates both the peripheral and central serotonergic tones to modulate energy balance remains to be determined. Given the consistent differences in energy expenditure between experimental groups across multiple days and the relatively shorter half-life of propranolol (~2–6 h) and ondansetron (~3–5 hours) [65,66,67], it is highly unlikely that the prior administration of these drugs confounded the subsequent effects of the dietary interventions on energy expenditure.

Rats fed with 16AA-Met decreased body weight, body fat mass, and body lean mass, with the weight and fat loss being more protracted (day 7–21) than 5AA and 5AA+Met (day 11–21). This is likely due to the increased energy expenditure and a reduction in the total amount of calories consumed, and is in line with other studies reporting that dietary methionine restriction promotes energy expenditure and hypophagia [13,30,32,33,39,40,68]. In 6-OHDA-treated rats, the reduction in body weight, body fat mass, and body lean mass of the 16AA-Met, 5AA, and 5AA+Met groups were less robust, with the responses being delayed and of lower magnitude than the vehicle-treated animals. This indicates that chemical sympathectomy is protective against body weight and composition loss in these groups, and that an enhanced sympathetic tone contributes to the body weight, fat, and lean loss of animals fed with the 16AA-Met or 5AA diets. The decreased body weight and body lean mass in 5AA rats are in accordance with our previous findings and other studies [8,14,18,19,20]. The lack of body composition differences between the 5AA and 5AA+Met diets suggests that a supplementation of methionine alone is insufficient to totally or partially reverse the metabolic responses of 5AA and is consistent with a lack of metabolic improvements with BCAA supplementation to low protein diets [29,44]. Given that dietary protein dilution [69] and methionine restriction [70] improve indices of metabolic health in humans, it remains to be determined whether methionine restriction promotes sympathetically driven energy expenditure in humans.

## 5. Conclusions

In summary, we provide evidence that methionine-restricted diets, in part, recapitulate the enhanced energy expenditure, as well as weight and lean loss resulting from total AA restriction, whereas supplementation of methionine alone to required levels was ineffective in fully or partially reversing the effects of total AA-restricted diets on energy balance. Although chemical sympathectomy, in part, negated the effects of total AA and methionine-restricted diets on measures of energy expenditure and body composition, the sympathetic system does not seem to be the only mediator of such effects. Further research is warranted to assess the role of other essential AA as well as neuroendocrine-related mechanisms engaged by AA restriction to modulate energy balance.

## Figures and Tables

**Table 1:** The composition of the diets ^1.^

	16AA	5AA	5AA+Met	16AA-Met
**Ingredients (g kg^−1^) ^1^**				
Corn starch	363.94	482.98	479.93	366.98
L-Alanine	4.57	1.52	1.52	4.57
L-Arginine	6.36	2.12	2.12	6.36
L-Aspartic acid	12.13	4.04	4.04	12.13
L-Cystine	3.68	1.23	1.23	3.68
L-Glutamic acid	36.10	12.03	12.03	36.10
Glycine	3.18	1.06	1.06	3.18
L-Histidine	4.57	1.52	1.52	4.57
L-Isoleucine	8.45	2.82	2.82	8.45
L-Leucine	15.31	5.10	5.10	15.31
L-Lysine	12.93	4.31	4.31	12.93
D,L-Methionine	4.57	1.52	4.57	1.52
L-Phenylalanine	8.75	2.92	2.92	8.75
L-Proline	20.38	6.79	6.79	20.38
L-Serine	9.64	3.21	3.21	9.64
L-Threonine	6.66	2.22	2.22	6.66
L-Tryptophan	2.09	0.70	0.70	2.09
L-Tyrosine	9.25	3.08	3.08	9.25
L-Valine	9.95	3.32	3.32	9.95
Sucrose	200.00	200.00	200.00	200.00
Corn oil	60.00	60.00	60.00	60.00
Lard	100.00	100.00	100.00	100.00
α-Cellulose	50.00	50.00	50.00	50.00
AIN-93-MX	35.00	35.00	35.00	35.00
AIN-93-VX	10.00	10.00	10.00	10.00
Choline bitartrate	2.50	2.50	2.50	2.50
**Composition**				
Protein (% kcal) ^2^	16.20%	5.40%	5.68%	15.92%
Carbohydrate (% kcal) ^2^	51.15%	61.95%	61.67%	51.43%
Fat (% kcal) ^2^	32.65%	32.65%	32.65%	32.65%
Energy density, kcal/g ^2^	4.41	4.41	4.41	4.41
Methionine (DL)% diet ^3^	0.46	0.15	0.46	0.15

^1^ The diets were prepared in-house following AIN-93 recommendations [48] and using individual amino acids (AA) and ingredients from Dyets Inc. (Bethlehem, PA, USA). 16AA, 16% amino acids; 5AA, 5% amino acids; 5AA+Met, 5% amino acids, methionine supplemented; 16AA-Met, 16% amino acids, methionine restricted. ^2^ The energy density of the nutrients was calculated from the estimated caloric values of protein, fat, and carbohydrate at 4, 9, and 4 kcal/g respectively. ^3^ Calculated as 100 × (Methionine in g/total amount of respective diet in g).

**Table 2:** The effect of dietary methionine restriction or supplementation on the food conversion rate, relative energy intake, and energy efficiency of the vehicle- and 6-OHDA-treated obesity-prone rats for 3 weeks ^1.^

			Time			*p*-Values		
Treatment	Variable	Diet	Week 1	Week 2	Week 3	Diet	Week	Diet × Week
Vehicle (V)	Food Conversion Rate ^2^ (kcal intake/kcal energy deposited)	V16AA	1.7 ± 0.3	2.2 ± 0.3	3.6 ± 0.3	<0.01	<0.01	<0.01
		V5AA	2.4 ± 0.3^a^	3.5 ± 0.3^a^	4.1 ± 0.4			
		V5AA+Met	2.4 ± 0.3^b^	3.2 ± 0.3^b^	5.4 ± 0.4			
		V16AA-Met	5.3 ± 0.4^ce^	2.3 ± 0.3^e^	3.5 ± 0.3			
	Relative Energy Intake ^3^ (kcal intake/100 g body weight)	V16AA	33.4 ± 0.7	29.1 ± 1.5	24.5 ± 1.4	0.08	<0.01	0.59
		V5AA	37.8 ± 2.0^a^	29.9 ± 1.5	28.1 ± 1.5			
		V5AA+Met	36.4 ± 1.3^b^	32.3 ± 2.1	26.5 ± 1.9			
		V16AA-Met	37.9 ± 4.8	35.9 ± 1.6^ce^	27.0 ± 1.8			
	Energy Efficiency ^4^ (kcal energy deposited/kcal intake)	V16AA	0.59 ± 0.02	0.47 ± 0.02	0.30 ± 0.02	<0.01	<0.01	<0.01
		V5AA	0.41 ± 0.02^a^	0.30 ± 0.02^a^	0.25 ± 0.03			
		V5AA+Met	0.42 ± 0.02^b^	0.35 ± 0.02^b^	0.20 ± 0.03			
		V16AA-Met	0.21 ± 0.03^ce^	0.44 ± 0.02^e^	0.29 ± 0.02			
6-OHDA (S)	Food Conversion Rate ^2^ (kcal intake/kcal energy deposited)	S16AA	1.3 ± 0.3	2.1 ± 0.3	3.3 ± 0.3	<0.01	<0.01	0.23
		S5AA	1.8 ± 0.3^a^	3.3 ± 0.3^a^	5.2 ± 0.3^a^			
		S5AA+Met	1.7 ± 0.3^b^	3.6 ± 0.3^b^	5.1 ± 0.3^b^			
		S16AA-Met	1.9 ± 0.3^c^	2.6 ± 0.3^ce^	4.5 ± 0.3^c^			
	Relative Energy Intake ^3^ (kcal intake/100 g body weight)	S16AA	32.4 ± 1.7	29.6 ± 3.1	24.8 ± 0.9	<0.01	<0.01	0.29
		S5AA	38.8 ± 1.7^a^	32.1 ± 2.6	25.8 ± 1.2			
		S5AA+Met	38.3 ± 1.3^b^	35.6 ± 1.3	29.1 ± 1.0^b^			
		S16AA-Met	31.5±1.8^e^	33.7 ± 3.3	23.4 ± 1.0			
	Energy Efficiency ^4^ (kcal energy deposited/kcal intake)	S16AA	0.77 ± 0.03	0.43 ± 0.03	0.31 ± 0.03	<0.01	<0.01	0.12
		S5AA	0.59 ± 0.03^a^	0.32 ± 0.03	0.20 ± 0.03^a^			
		S5AA+Met	0.61 ± 0.03^b^	0.31 ± 0.03	0.21 ± 0.03^b^			
		S16AA-Met	0.59 ± 0.03^c^	0.32 ± 0.03	0.20 ± 0.03^c^			

^1^ The data for the antagonists’ administration days (i.e., days 9, 12, 14, and 16) were not included in the table or data analysis. The *p*-values for the overall model effect for treatment, diet, week, treatment × diet, treatment × week, diet × week, and treatment × diet × week for the food conversion rate were 0.06, <0.01, <0.01, 0.36, <0.01, <0.01, and <0.01, respectively; for relative energy intake were 0.64, <0.01, <0.01, 0.08, 0.74, 0.29, and 0.93, respectively; and for energy efficiency were <0.01, <0.01, <0.01, 0.71, <0.01, <0.01, and 0.06, respectively. V16AA, vehicle-treated 16% amino acid diet; V-5AA, vehicle-treated 5% amino acid diet; V5AA+Met, vehicle-treated 5% amino acid and methionine-supplemented diet; V16AA-Met, vehicle-treated 16% amino acid and methionine-restricted diet. S16AA, sympathectomized 16% amino acid diet; S5AA, sympathectomized 5% amino acid diet; S5AA+Met, sympathectomized 5% amino acid and methionine-supplemented diet; and S16AA-Met, sympathectomized 16% amino acid and methionine-restricted diet. Within the columns for each variable, ^a^ *p* ≤ 0.05 16AA vs. 5AA, ^b^ *p* ≤ 0.05 16AA vs. 5AA+Met, ^c^ *p* ≤ 0.05 16AA vs. 16AA-Met, and ^e^ *p* ≤ 0.05 5AA vs. 16AA-Met. The values are the mean ± SEM, *n* = 7–8. ^2^ Calculated as the accumulated weekly energy intake divided by the weekly energy deposited (sum of lean mass (g) × 4 kcal/g and fat mass (g) × 11.1 kcal/g) [51]. ^3^ Calculated as the accumulated weekly energy intake divided by 100 g of body weight. ^4^ Calculated as the accumulated weekly energy deposited (sum of lean mass (g) × 4 kcal/g and fat mass (g) × 11.1 kcal/g) divided by the weekly energy intake [51].

**Table 3:** The effect of saline or propranolol injections on the area under the curve for energy intake and energy expenditure of vehicle- and 6-OHDA-treated obesity-prone rats fed diets supplemented with methionine or restricted in methionine ^1^.

Treatment	Diet	Time	Drug	Energy Balance	
				Energy Intake kcal × h	Energy Expenditure (kcal/ h) × h
Vehicle (V)	V16AA	Dark	Saline	76 ± 6	36 ± 1
			Propranolol	62 ± 6	32 ± 1 *
		Light	Saline	16 ± 2	21 ± 1
			Propranolol	17 ± 2	20 ± 1
	V5AA	Dark	Saline	73 ± 3	39 ± 1
			Propranolol	68 ± 7	33 ± 1 *
		Light	Saline	16 ± 1	23 ± 1
			Propranolol	13 ± 2	21 ± 1
	V5AA+Met	Dark	Saline	74 ± 4	37 ± 0.9
			Propranolol	56 ± 2 *	33 ± 0.9 *
		Light	Saline	11 ± 2	20 ± 0.9
			Propranolol	10 ± 2	20 ± 0.9
	V16AA-Met	Dark	Saline	79 ± 9	30 ± 1
			Propranolol	81 ± 10	28 ± 1
		Light	Saline	10 ± 2	19 ± 1
			Propranolol	16 ± 2	18 ± 1
6-OHDA (S)	S16AA	Dark	Saline	74 ± 3	35 ± 1.8
			Propranolol	69 ± 8	31 ± 1.8
		Light	Saline	16 ± 1	19 ± 1.8
			Propranolol	13 ± 2	18 ± 1.8
	S5AA	Dark	Saline	65 ± 4	37 ± 1.7
			Propranolol	53 ± 3 *	28 ± 1.7 *
		Light	Saline	21 ± 4	21 ± 1.7
			Propranolol	12 ± 3	18 ± 1.7
	S5AA+Met	Dark	Saline	76 ± 4	37 ± 1.7
			Propranolol	55 ± 4 *	30 ± 1.7 *
		Light	Saline	14 ± 2	20 ± 1.7
			Propranolol	12 ± 3	20 ± 1.7
	S16AA-Met	Dark	Saline	69 ± 4	35 ± 1.7
			Propranolol	48 ± 5 *	29 ± 1.7
		Light	Saline	11 ± 2	19 ± 1.7
			Propranolol	11 ± 2	18 ± 1.7

^1^ The area under the curve was calculated for energy intake and energy expenditure over 20 hours with 10 hours for the dark and 10 hours for the light cycles, respectively. All rats were injected with saline or drug (i.e., propranolol, 10 mg/kg, SC) between days 9–16. The *p*-values for the overall model effect for treatment, diet, drug, and treatment × diet for energy intake were 0.53, 0.72, 0.19, and 0.89, respectively, and for energy expenditure were 0.04, <0.01, <0.01, and 0.02, respectively. The nonsignificant (*p* > 0.05) interactions are not shown. V16AA, vehicle-treated 16% amino acid diet; V5AA, vehicle-treated 5% amino acid diet; V5AA+Met, vehicle-treated 5% amino acids and methionine-supplemented diet; V16AA-Met, vehicle-treated 16% amino acid and methionine-restricted diet; S16AA, sympathectomized 16% amino acid diet; S-5AA, sympathectomized 5% amino acid diet; S5AA+Met, sympathectomized 5% amino acids and methionine-supplemented diet; S16AA-Met, sympathectomized 16% amino acid and methionine-restricted diet. Within the columns, * *p* < 0.05 saline vs. propranolol within each treatment, diet, and time. The values are the mean ± SEM, *n* = 7–8.

**Table 4:** The effect of saline or ondansetron injections on the area under the curve for energy intake and energy expenditure of the vehicle- and 6-OHDA-treated obesity-prone rats fed with diets supplemented with methionine or restricted of methionine ^1.^

Treatment	Diet	Time	Drug	Energy Balance	
				Energy Intake	Energy Expenditure
				kcal × h	(kcal/ h) × h
Vehicle (V)	V-16AA	Dark	Saline	63 ± 3	37 ± 3
			Ondansetron	74 ± 5	34 ± 3
		Light	Saline	20 ± 3	21 ± 3
			Ondansetron	14 ± 1 *	20 ± 3
	V-5AA	Dark	Saline	56 ± 5	30 ± 2
			Ondansetron	69 ± 3	38 ± 3
		Light	Saline	19 ± 3	17 ± 2
			Ondansetron	17 ± 1	22 ± 3
	V-5AA+Met	Dark	Saline	60 ± 3	41 ± 2
			Ondansetron	68 ± 4	37 ± 2
		Light	Saline	16 ± 3	21 ± 2
			Ondansetron	10 ± 1	20 ± 2
	V-16AA-Met	Dark	Saline	69 ± 7	41 ± 2
			Ondansetron	76 ± 4	33 ± 2
		Light	Saline	20 ± 3	23 ± 2
			Ondansetron	10 ± 2 *	19 ± 2
6-OHDA (S)	S-16AA	Dark	Saline	67 ± 3	38 ± 1
			Ondansetron	65 ± 5	31 ± 1 *
		Light	Saline	17 ± 2	21 ± 1
			Ondansetron	14 ± 1	18 ± 1
	S-5AA	Dark	Saline	70 ± 7	41 ± 1
			Ondansetron	61 ± 3	34 ± 1 *
		Light	Saline	14 ± 3	22 ± 1
			Ondansetron	15 ± 1	20 ± 1
	S-5AA+Met	Dark	Saline	69 ± 4	40 ± 1
			Ondansetron	73 ± 4	34 ± 1 *
		Light	Saline	15 ± 2	21 ± 1
			Ondansetron	12 ± 2	19 ± 1
	S-16AA-Met	Dark	Saline	55 ± 3	37 ± 1
			Ondansetron	69 ± 6	31 ± 1 *
		Light	Saline	16 ± 2	21 ± 1
			Ondansetron	12 ± 2	18 ± 1

^1^ The area under the curve was calculated for energy intake and energy expenditure over 20 h with 10 hours for the dark and 10 h for the light cycles, respectively. All rats were injected with saline or a drug (i.e., ondansetron, 1 mg/kg; IP) between days 9–16. The *p*-values for the overall model effect for treatment, diet, drug, and treatment × diet for energy intake were 0.91, 0.91, 0.57, and 0.96, respectively. The *p*-values for the overall model effect for treatment, diet, drug, diet × drug, drug × treatment, and diet × drug × treatment for energy expenditure were 0.56, 0.36, <0.01, 0.01, 0.02, and <0.01, respectively. The nonsignificant (*p* > 0.05) interactions are not shown. V16AA, vehicle-treated 16% amino acid diet; V5AA, vehicle-treated 5% amino acid diet; V5AA+Met, vehicle-treated 5% amino acids and methionine-supplemented diet; V16AA-Met, vehicle-treated 16% amino acid and methionine-restricted diet; S16AA, sympathectomized 16% amino acid diet; S-5AA, sympathectomized 5% amino acid diet; S5AA+Met, sympathectomized 5% amino acids and methionine-supplemented diet; S16AA-Met, sympathectomized 16% amino acid and methionine-restricted diet. Within the columns, * *p <* 0.05 saline vs. ondansetron within each treatment, diet, and time. The values are the mean ± SEM, *n* = 7–8.
